# iTRAQ-based quantitative analysis reveals proteomic changes in Chinese cabbage (*Brassica rapa* L.) in response to *Plasmodiophora brassicae* infection

**DOI:** 10.1038/s41598-019-48608-0

**Published:** 2019-08-19

**Authors:** Mei Lan, Guoliang Li, Jingfeng Hu, Hongli Yang, Liqin Zhang, Xuezhong Xu, Jiajia Liu, Jiangming He, Rifei Sun

**Affiliations:** 1 Institute of Horticultural Crops, Yunnan Academy of Agricultural Sciences, Yunnan Branch of the National Vegetable Improvement Center, Kunming, 650205 China; 2 Institute of Vegetables and Flowers, Chinese Academy of Agricultural Sciences, Zhongguancun, Nandajie No. 12, Haidian District, Beijing, 100081 China; 3 Yunnan University of Chinese Medicine, Kunming, 650500 China

**Keywords:** Protein-protein interaction networks, Plant stress responses

## Abstract

Clubroot disease is one of the major diseases affecting *Brassica* crops, especially Chinese cabbage (*Brassica rapa* L. *ssp*. *pekinensis*), which is known to be highly susceptible to the disease. In this study, the obligate biotrophic protist *Plasmodiophora brassicae* Woronin was used to infect the roots of Chinese cabbage seedlings. The disease symptoms were noticeable at 28 and 35 days after inoculation (DAI) in the susceptible (CM) line. Using isobaric tags for relative and absolute quantitation (iTRAQ) analysis, a total of 5,003 proteins of differential abundance were identified in the resistant/susceptible lines, which could be quantitated by dipeptide or polypeptide segments. Gene ontology (GO) analysis indicated that the differentially expressed proteins (DEPs) between the susceptible (CM) and resistant (CCR) lines were associated with the glutathione transferase activity pathway, which could catalyze the combination of glutathione and other electrophilic compounds to protect plants from disease. In addition, the Kyoto Encyclopedia of Genes and Genomes (KEGG) analysis revealed that the DEPs may be significantly enriched cytokinin signaling or arginine biosynthesis pathways, both of which are responses to stimuli and are plant defense reactions. The cytokinins may facilitate cell division in the shoot, resulting in the hypertrophy and formation of galls and the presentation of typical clubroot symptoms. In this study, the proteomic results provide a new perspective for creating germplasm resistance to *P*. *brassicae*, as well as a genetic basis for breeding to improve Chinese cabbage.

## Introduction

Chinese cabbage (*Brassica rapa* L. *ssp*. *pekinensis*) is an important cruciferous vegetable that is consumed worldwide. It is also highly susceptible to infection by the obligate biotrophic protist *Plasmodiophora brassicae* Woron, known as clubroot^1–5^. Approximately, 3.2–4.0 million ha of cruciferous crops are infected by *P*. *brassicae*, accounting for more than 33% of the cultivation area, leading to 20–30% annual yield losses^6,7^. Globally, clubroot is the most damaging disease of brassica crops and occurs in more than 60 countries, where the yield losses are generally in the range of 10–15%^3^, but can reach higher than 80%^8–10^. The incidence of clubroot infection in *Brassica rapa* has been increasing around the world, especially in China. According to the reactions on the Williams differentials, the *P*. *brassicae* pathotype 4 is the most common strain in China, and is responsible for nearly 80% of all infections^11^.

*P*. *brassicae* is a disease typically associated with warm and wet soil^4^. Clubroot infection begins when resting spores germinate and motile zoospores swim through soil or water to the host roots; a process is thought to be stimulated by root exudates^12,13^. When resting spores are in soil, they have a half-life of approximately 4 years can remain viable for many years^14^. During the primary infection stage, the zoospores infect root hairs, where they propagate and proceed to spread and form secondary infections in root cells^10,15^. Pathogen development in the root cortical cells leads to changes in the root hormone balance, which results in hypertrophy and the formation of galls, a typical clubroot symptom^16,17^. The infected roots can produce resting spores that can survive for so long that standard strategies, such as antimicrobial compounds and crop rotations, cannot eliminate the pathogen^2,18^. To obtain durable and broad-spectrum resistance to *P*. *brassicae* in Chinese cabbage, resistant proteins and genes must be identified and used in the development of resistant varieties.

Many studies have been conducted to increase our knowledge of *P*. *brassicae*, including its biological characteristics^19^, physiological races^20,21^, and the quantitative trait loci (QTLs)^22^, but the mapping and cloning of resistance genes, such as *Crr1*/2/4^23^, *Crr3*^24^, *CRc*/*CRk*^25^, *CRa*^26^, *CRb*^*kato*^ ^27^, *PbBa3*.1/3^28^, *CRb*^29^, *Rcr1*^30^, *Rcr2*^31^, *Rcr4*/8/9^32^, *CrrA5*^33^, *CRd*^34^, and *CRs*^35^, which would increase our understanding of the interaction between *B*. *rapa* and *P*. *brassicae*. Most of the resistant genotypes that have been identified appear to have pathotype-specific resistance, although some have shown a broader resistance spectrum^36,37^. In recent years, using transcriptome analysis of broccoli, wild cabbage^38^, and *A*. *thaliana*^39^, the defense mechanisms of *B*. *rapa* to *P*. *brassicae* have been described, but a proteomic analysis of the response of *B*. *rapa* to *P*. *brassicae* has only been reported a limited number of times. The few existing reports include a shotgun label-free proteomic analysis of clubroot (*Plasmodiophora brassicae*) resistance conferred by the gene *Rcr1* in *Brassica rapa*^40^, iTRAQ analysis of proteins profiles during the secondary stage of infection of *Plasmodiophora brassicae* in Chinese cabbage^41^, and proteomic analysis of the interaction between *Plasmodiophora brassicae* and Chinese cabbage at the initial infection stage^42^.

Plants initiate specific defense responses by recognizing particular signals from damaged cells, and when infected by pathogens during active growth, plants can implement efficient and systemic defense mechanisms^43,44^. The molecular mechanisms resulting from environmental stress tolerance are very complex in plants^45^. Stress-transcriptomic and -proteomic analyses are two effective approaches for studying gene and protein expression, respectively, in the biological processes under stress^46–48^. Using the *A*. *thaliana*-*P*. *brassicae* interaction pathosystem, the proteins in roots and stems from infected and non-infected plants have been analyzed^49^. Compared with non-infected plants, proteins associated with cell defense, metabolism, and cell differentiation showed a greater abundance in infected plants. Given the critical role of proteins in almost all cellular functions, proteomic analyses, which provide comprehensive qualitative and quantitative information for hundreds to thousands of proteins, have become an important tool for studying biological processes^50^. In particular, the modern proteomics technique, isobaric tags for relative and absolute quantitation (iTRAQ), may provide an efficient approach for explaining specific protein functions^51^.

High-throughput profiling of protein species, with the advantages of dynamic landscapes, deep coverage, and high resolution, is a powerful method for analyzing changes in intricate processes^52^. In this study, Chinese cabbage lines with both resistance and ssusceptibility to *P*. *brassicae* were inoculated, and plants that developed galls underwent iTRAQ-based proteomic analysis. The proteins associated with resistance to *P*. *brassicae* were identified, and resistance and defense mechanisms in response to *P*. *brassicae* were explored at the proteomic level. The results of this study may lay the foundation for the development of germplasm innovation, as well as for the development of antibody resistance to *P*. *brassicae* in Chinese cabbage.

## Results

### Phenotype analysis after inoculation

**Figure 1:**
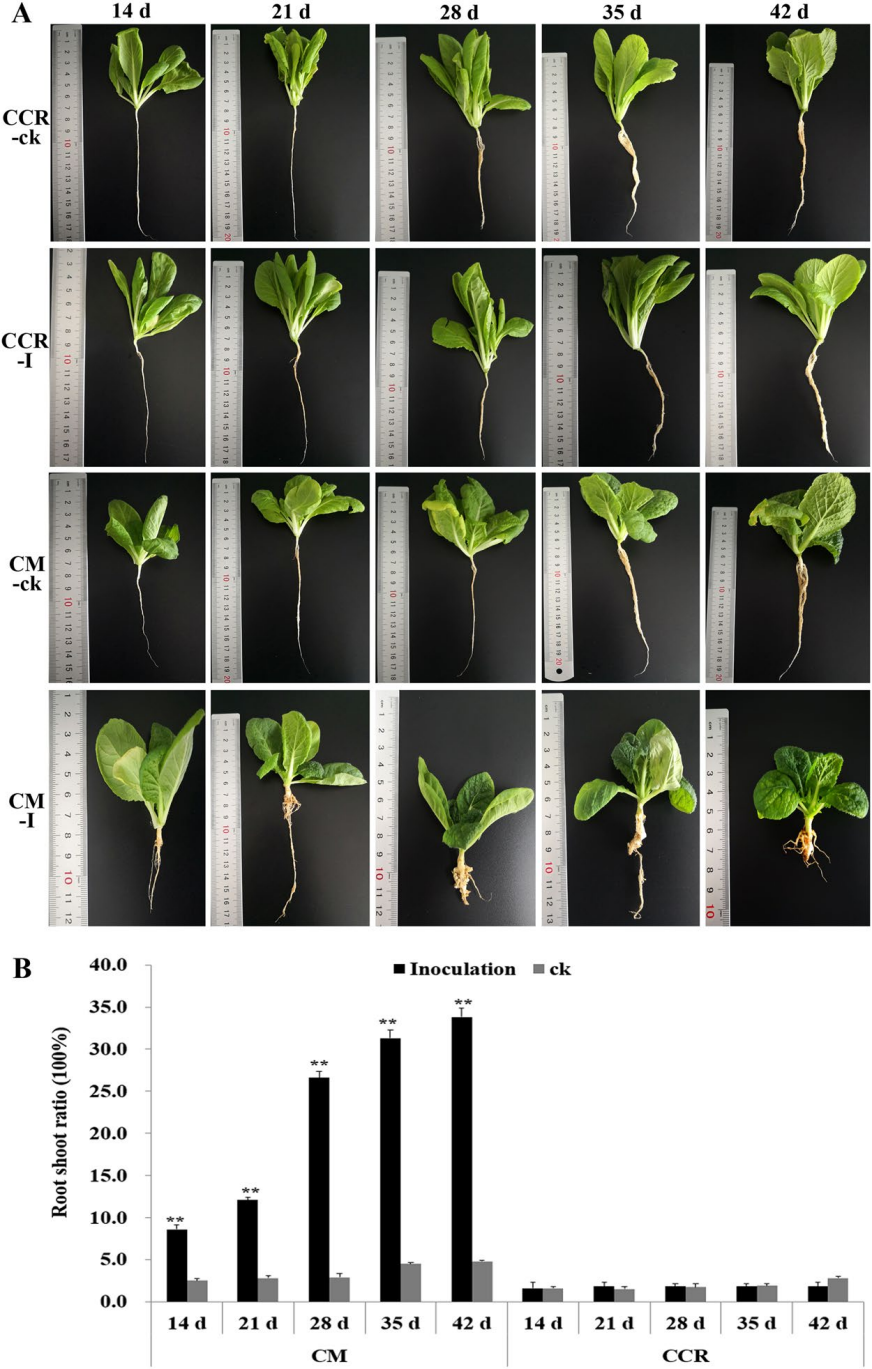
Analysis of a swelled root in the *Brassica rapa* lines CCR and CM after inoculation with *Plasmodiophora brassicae*. (**A**) 14, 21, 28, 35, and 42 days after inoculation with *P*. *brassicae*. CCR-ck and CM-ck were two controls, CCR-I denotes CCR-inoculation, and CM-I denotes CM-inoculation. (**B**) The root-shoot ratios of CCR-ck, CCR-I, CM-ck, and CM-I at five periods in time.

The phenotypes of CCR and CM were investigated at 14, 21, 28, 35, and 42 days after inoculating (DAI) *P*. *brassicae* (Fig. 1A). The roots of CCR-ck and CM-ck did not form root galls from 14 to 42 DAI, indicating that the controls were reliable, and the resistant lines were resistant to *P*. *brassicae*. Visual changes to the root of CM-I clearly showed that the CM line was susceptible to *P*. *brassicae*, and there were an increasing number of root galls from 14 to 42 DAI. In addition, the root-shoot ratio for CCR-ck, CCR-I, CM-ck, and CM-I was consistent with the phenotype investigations (Fig. 1B). The root-shoot ratios of CCR-ck, CCR-I, and CM-ck were stable from 14 to 42 DAI, and the average for CM-ck was higher than CCR-ck, indicating that the root swelling of the susceptible line (CM line) was initially more pronounced than in the resistant line (CCR line) at the same stage. The root-shoot ratio of CM-I rapidly increased from 14 to 42 DAI and was much higher than CCR-ck, CCR-I, and CM-ck. Additionally, the roots of the CM line were observed to quickly swelled under *P*. *brassicae* stimulation. Compared with 21 DAI, the root of CM-I greatly varied at 28 DAI and compared with 42 DAI, where the roots of CM-I at 35 DAI tended to be stable (Fig. 1A,B). Therefore, these two periods (28 and 35 DAI) were chosen for proteome analyses.

### Overview of the unique proteins identified using iTRAQ data

**Figure 2:**
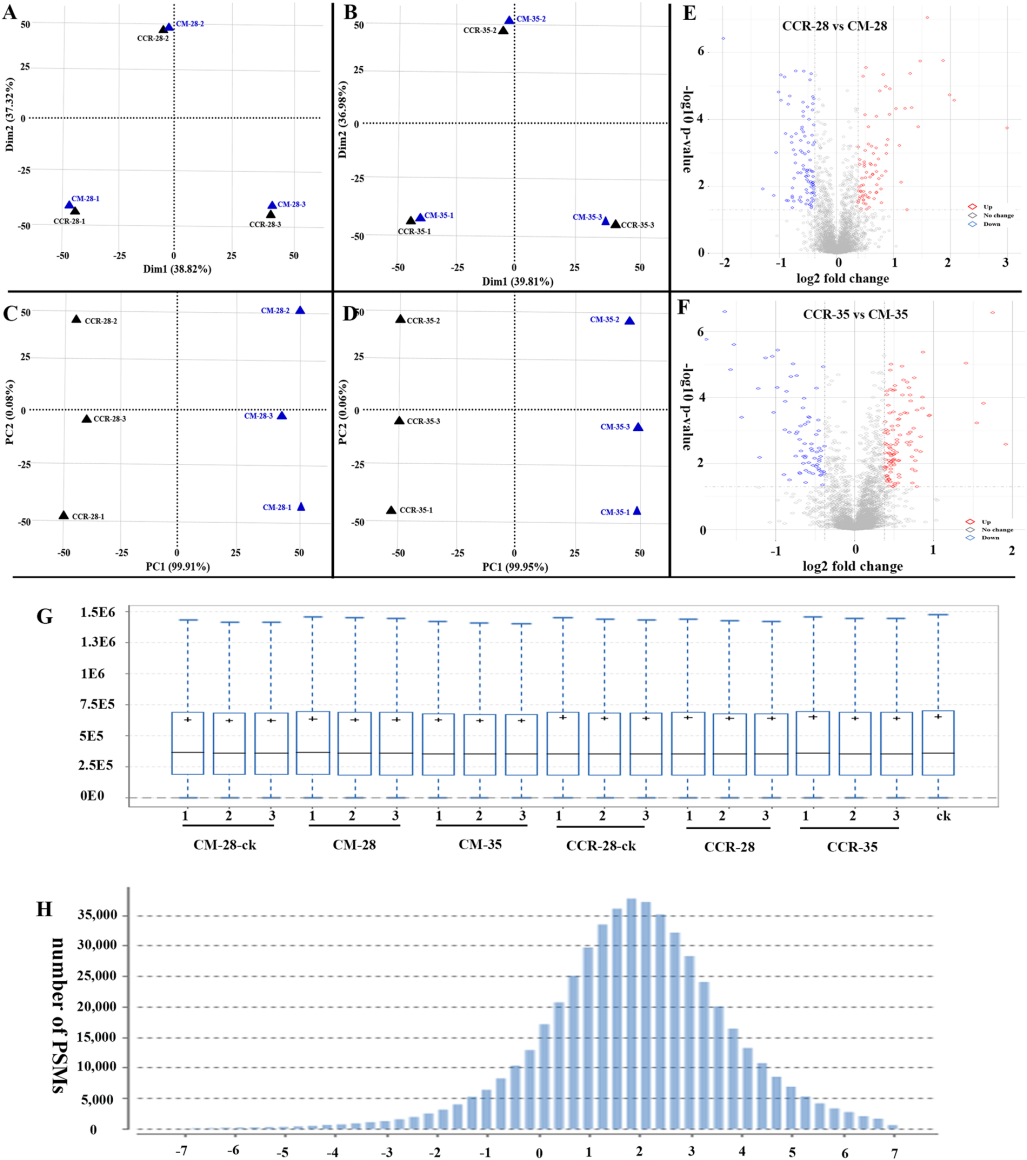
Overview of the unique proteins identified using iTRAQ data. PCA was used to analyze the main proteins in (**A**) CCR-28/CM-28 and (**B**) CCR-35/CM-35. PLS-DA was used to analyze the main proteins in (**C**) CCR-28/CM-28 and (**D**) CCR-35/CM-35. (**E**) Analysis of the DEPs between CCR-28 and CM-28 using volcano figures. (**F**) Analysis of the DEPs between CCR-35 and CM-35 using volcano figures. (**G**) Signal normalization of the signal boxes for each channel. The figure can be used to understand the normalization effect through the median of each channel. (**H**) Quality control of quality precision. The abscissa is the mass accuracy distribution of the mass spectrometry detection, and the ordinate is the corresponding distribution of the number of matching results.

The proteomes of the six samples (CM-28-ck, CM-28, CM-35, CCR-28-ck, CCR-28, and CCR-35) were analyzed using the iTRAQ). Principal component analysis (PCA) was used for the main proteins in the CCR and CM samples, and it showed a clear separation between CCR and CM, with satisfactory sample repeatability using three repetitions (Fig. 2A,B). Partial least squares discrimination analysis (PLS-DA) results were consistent with the PCA (Fig. 2C,D). Using *B*. *rapa* (Chiifu-401) as the reference, the DEPs were analyzed and volcano figures were created (Fig. 2E). Between CCR-28 and CM-28, there were 131 upregulated DEPs and 110 downregulated DEPs. Similarly, between CCR-35 and CM-35, there were 75 upregulated DEPs and 61 downregulated DEPs (Fig. 2F).

Using mass spectrometry qualitative and quantitative results visualization to control quality, it was determined that the sample separation was normal (Fig. S1), and the effective separation time was 20–110 min. The effective separation time accounted for 80%, indicating that no polyethylene glycol (PEG), salt, or other exogenous pollution were present. Normalization analysis showed that there were no abnormal fluctuations in median or box plot distributions for each channel, indicating that the mass spectral data were normalized (Fig. 2G). The signal concentrated out of the peak, indicating that the chromatographic separation conditions were ideal and the width of the spectrum peak was approximately half a minute, which was also ideal. Mass quality control analysis showed that above 10,000, peptide-spectrum matches (PSMs) were between 0 and 4 ppm, indicating that the axis of the mass spectrum was stable and the mass accuracy was very high (Fig. 2H).

### Identification and analysis of the DEPs

**Figure 3:**
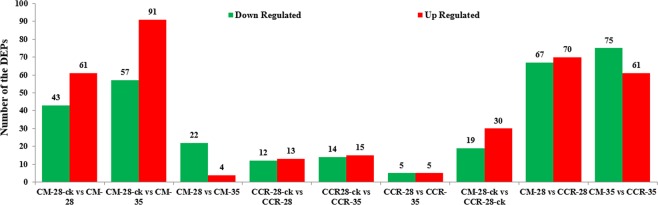
Identification of analysis of the DEPs in the nine sample combinations: CM-28-ck/CM-28, CM-28-ck/CM-35, CM-28/CM-35, CCR-28-ck/CCR-28, CCR-28-ck/CCR-35, CCR-28/CCR-35, CM-28-ck/CCR-28-ck, CM-28/CCR-28, and CM-35/CCR-35.

PEAKS DB software^53^ was used for quantitative analysis and manual confirmation. Based on the reference sequence of *B*. *rapa* (Chiifu-401), 5,003 proteins were successfully matched and were quantified by the dipeptide or polypeptide segments in BLAST. 61 DEPs were upregulated and 43 were downregulated in, and 91 DEPs upregulated and 57 DEPs downregulated in CM-28 compared with CM-28-ck. However, the comparisons between CM-28/CM-35, CCR-28-ck/CCR-28, CCR-28-ck/CCR-35, CCR-28/CCR-35, and CM-28-ck/CCR-28-ck showed fewer DEPs (4 upregulated/22 downregulated, 13 upregulated/12 downregulated, 15 upregulated/14 downregulated, 5 upregulated/5 downregulated, and 30 upregulated/19 downregulated, respectively) (Fig. 3). There were 137 DEPs between CCR-28 and CM-28, including 70 upregulated and 67 downregulated, and there were 136 DEPs between CCR-35 and CM-35, including 61 upregulated and 75 downregulated (Fig. 3). Almost all of the DEPs were distributed during the early and middle stages following inoculation with *P*. *brassicae*, and protein abundance changes at the peak of infestation were limited. This indicated that the effect of *P*. *brassicae* on the growth of Chinese cabbage was mainly due to its infestation pre-intermediate stages.

**Figure 4:**
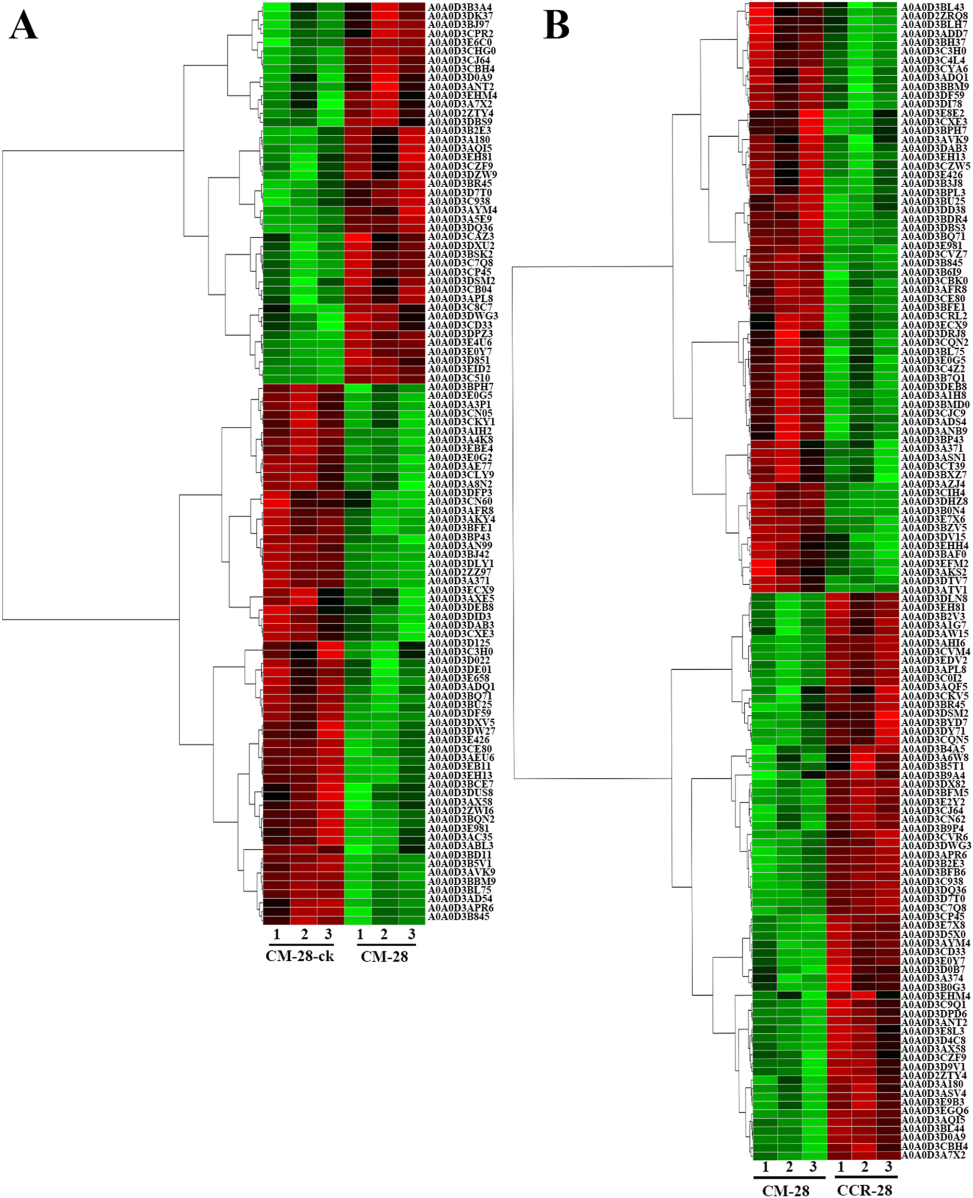
The hierarchical clustering and metabolic pathway analysis of the DEPs in the comparisons of CM-28-ck/CM-28 and CM-28/CCR-28.

Through hierarchical clustering analysis, the homologous proteins were classified as belonging to the same cluster in the comparison of nine combinations. The hierarchical cluster result of CM-28-ck and CM-28 showed that there were 104 DEPs, including 61 DEPs upregulated and 43 DEPs downregulated (Fig. 4A). There were 137 DEPs between CM-28 and CCR-28, including 70 upregulated and 67 downregulated (Fig. 4B). The hierarchical cluster analysis of other combinations is shown in Figs S2–4.

In the comparison of CM-28-ck/CM-28, the protein (stress responsive alpha-beta barrel domain) was clearly increasing and could regulate the metabolic pathway^54^, when the plant faced external stress. In the comparison of CM-28/CCR-28, the stress-responsive alpha-beta barrel domain protein and the early nodulin protein were clearly upregulated, and the early nodulin protein could lead to changes in the root hormone balance that would result in hypertrophy and formation of galls, which are typical clubroot symptoms^16,17^. In the comparison of CM-35/CCR-35, the pectinesterase inhibitor protein and the early nodulin protein were upregulated, and the pectinesterase inhibitor protein could inhibit the cell wall formation and facilitate the vacuole formation, which can accumulate and result in root hypertrophy^55^. In the other comparison combinations, the proteins (zinc finger, water chloroplastic, and glycoside hydrolase) were upregulated at different levels. Differential expression of up- and down-regulated glycoside hydrolase may be in response to phytohormone treatments, pathogen responsiveness, and abiotic stresses^56^.

### Gene ontology (GO) enrichment analysis and classification of the DEPs

**Figure 5:**
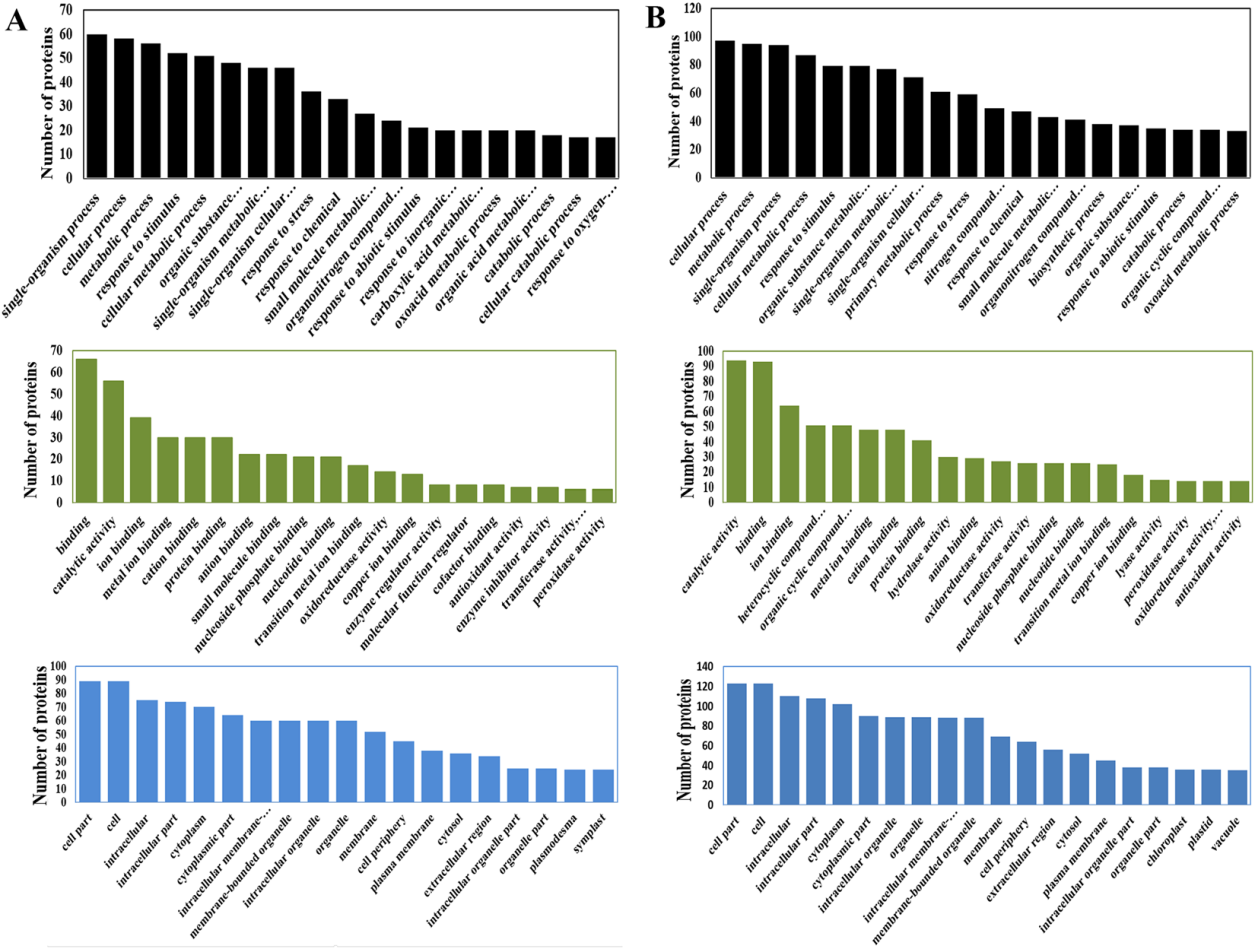
The DEP classification analysis from three GO categories in the comparison of (**A**) CM-28-ck/CM-28 and (**B**) CM-28/CCR-28, including: biological processes, cellular components, and molecular functions. Black bars represent the biological process, green bars represent the molecular function, and blue bars represent the cellular component.

The iTRAQ-based proteomics technology analysis showed the differences between clubroot-resistant and -susceptible varieties of Chinese cabbage in response to *P*. *brassicae*. These DEPs were classified into three GO categories: biological processes, cellular components, and molecular functions (Fig. 5A,B). For biological processes, in the comparison of CM-28-ck/CM-28, the protein with the largest proportion was the single-organism process protein. In the comparison of CM-28-ck/CM-35, CM-28/CCR-28, and CM-35/CCR-35, it was the cellular process protein. In the comparison of CM-28-ck/CM-28, CM-28-ck/CM-35, CM-28/CCR-28, and CM-35/CCR-35, the most predominant in cellular components was the cell part protein. In molecular function, in the comparison of CM-28-ck/CM-28, CM-28/CCR-28, and CM-35/CCR-35, the largest category was the catalytic activity protein, but in the comparison of CM-28-ck/CM-35 the largest was the binding protein. Between CM-28-ck and CM-28, there were 349 DEPs in the biological process category, 102 DEPs in the molecular function category, and 65 DEPs in the cellular component category. In the biological process category, the DEPs mainly involved the pathways of oxidoreductase activity, antioxidant activity, phosphatidylinositol-3,5-bisphosphate binding, glutathione transferase activity, phosphotransferase activity, and peroxidase activity. In the molecular function category, the DEPs were primarily associated with copper ion binding, glutathione transferase activity, and antioxidant activity. In the cellular component category, the DEPs were related to glutathione transferase activity, cytosol, extracellular region, plasmodesma, symplast, and cell periphery.

**Figure 6:**
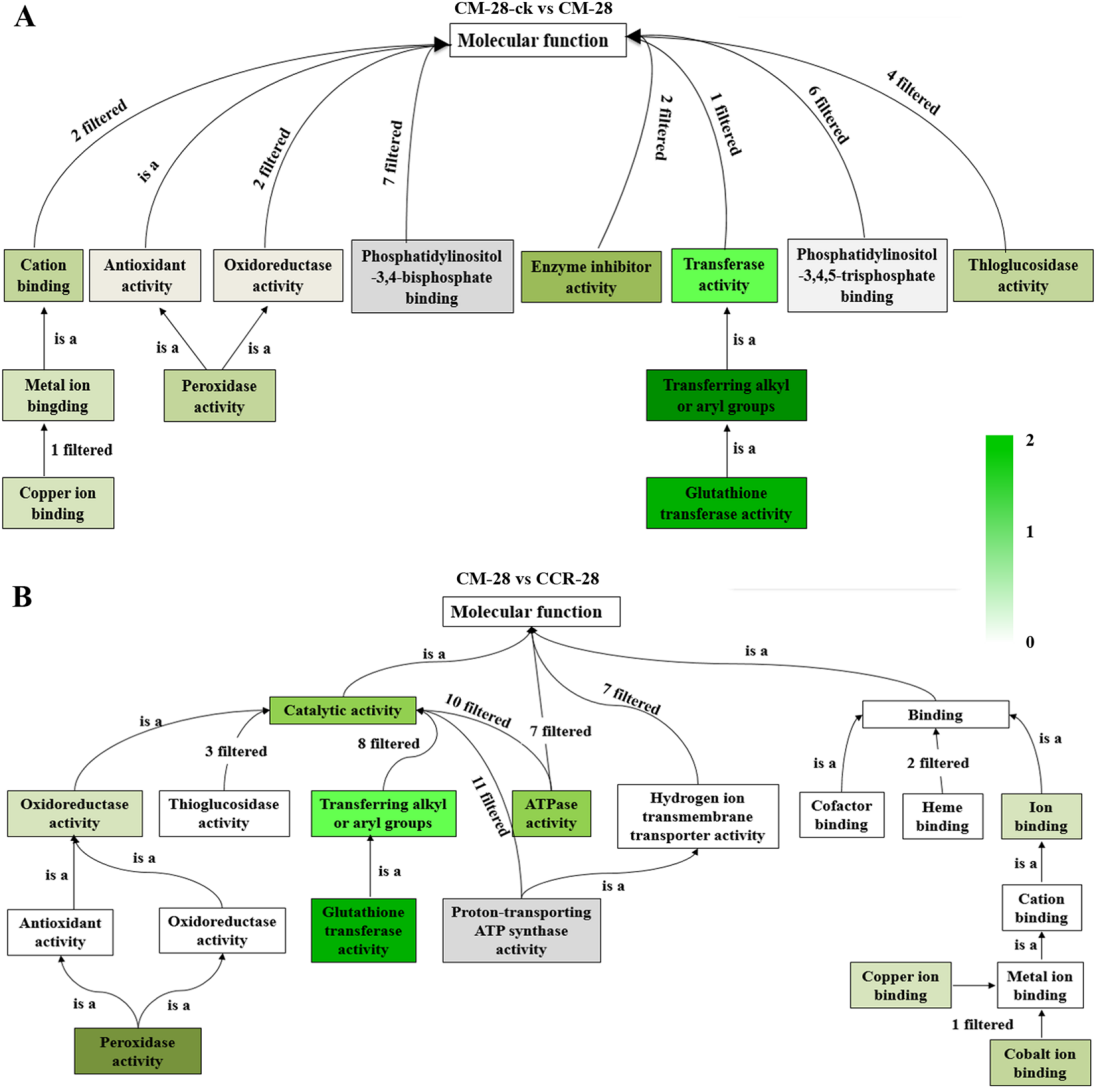
The DEP analysis from the glutathione transferase activity pathway in the comparison of (**A**) CM-28-ck/CM-28 and (**B**) CM-28/CCR-28. Green represents the number of DEPs in the comparison, and “is a” represents a containment relationship.

All three processes focused on the glutathione transferase activity pathway. In the comparison of CM-28-ck/CM-28 and CM-28/CCR-28, the glutathione transferase activity showed upregulation for both (Fig. 6A,B). Glutathione transferase is important in response to disease infection, which can catalyze the combination of glutathione and other electrophilic compounds to protect plants against germs^57^. In the current study, the Chinese cabbage lines inoculated with *P*. *brassicae* were stimulated to generate glutathione transferase, which would reduce the damage caused by *P*. *brassicae*. Indole-3-acetic acid (IAA) has been shown to promote the development of clubroot disease, including an increase in the number and size of galls^58^. High-affinity glutathione conjugates are also of special interest, as they appear to be able to very effectively uncouple auxin signaling from the IAA concentration^59^.

### Kyoto encyclopedia of genes and genomes (KEGG) pathway analysis

KEGG analysis was used to classify DEPs, and found that that the DEPs in CM-28 were significantly enriched for arginine biosynthesis and gluthathione, tryptophan, alanine, aspartate, and glutamate metabolism compared to CM-28-ck. The DEPs in CCR-28 were significantly enriched for phenylalanine and tyrosine metabolism and the biosynthesis of tropane, piperidine, and isoquinoline, and pyridine alkaloids. In the KEGG pathway of CCR-28-ck compared with CCR-28, three DEPs were involved in the phenylalanine metabolism pathway: calcium-dependent kinase 3, pyridoxal 5-phosphate synthase-like subunit, and calcium-transporting ATPase plasma membrane. Additionally, calcium-transporting ATPase plasma membrane and the pyridoxal 5-phosphate synthase-like subunit were involved in tyrosine metabolism and the biosynthesis of tropane, piperidine, and isoquinoline, and pyridine alkaloids.

**Figure 7:**
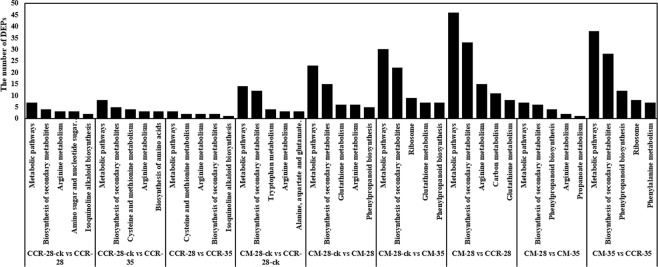
The number of DEPs from the nine comparisons obtained by KEGG analysis.

**Figure 8:**
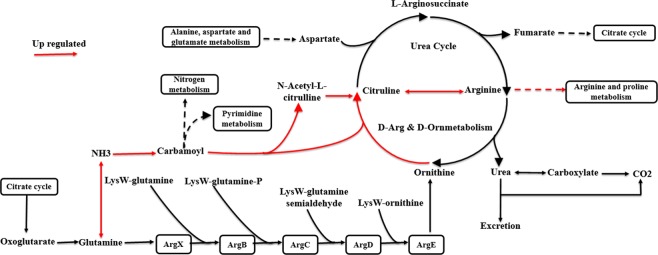
Analyses of the arginine metabolism pathway for the purpose of explaining the DEPs in the arginine biosynthesis process.

The expression analysis of the proteomes of CM-28-ck and CCR-28-ck revealed that the DEPs were mainly concentrated on the biosynthesis pathways of tyrosine, taurine, hypotaurine, butyric acid, and isoquinoline alkaloids. Based on the number of proteins classified in the same categories, the metabolic pathway occupied the largest proportion of the nine comparisons of lines, and the biosynthesis of secondary metabolism followed as having the second most proportion. Amino acid biosynthesis was greatly influenced, including arginine and glutathione metabolism (Fig. 7), and the arginine metabolism pathway was upregulated from glutamine to arginine generation. The pathway included the citrate cycle and urea cycle (Fig. 8), which are significant steps in the process of plant photosynthesis and respiration.

## Discussion

Proteomic analysis provides a large amount of information regarding the individual proteins that are involved in specific biological responses. Our iTRAQ-based proteomic data showed that the infected and control Chinese cabbage lines exhibited physiological and molecular changes in the pre-intermediate phases of infection from *P*. *brassicae*. In a previous study, protein samples extracted from *B*. *oleracea* roots 4 weeks after inoculation with *P*. *brassicae* showed that several proteins that are typically present in healthy roots were absent or strongly reduced in the infected roots exhibiting clubrooting systems^60^. In another study, the examination of *B*. *rapa* root protein patterns in compatible and incompatible interactions indicated that pathogenesis-related proteins were involved in the susceptible response^61^. The protein profiles of *Arabidopsis* roots 4 days after inoculation with *P*. *brassicae*, 12% of the visualized proteins had an altered abundance in infected plants compared with noninfected controls, including proteins involved in metabolism, cell differentiation, defense, and detoxification^62^.

Plant growth factors, including cytokinin, auxin, ethylene, abscisic acid, jasmonic acid, and salicylic acid, were regulated during infection with clubroot disease in other studies^17,49,62–67^. IAA had been involved in gall formation during the late-stages of infection and increased cytokinin levels in coordination with the development with clubroot disease symptoms, and IAA has also increased cell division during the beginning of club formation^17^. Plasmodia synthesize cytokinin has also been found to induce host cell division during clubroot disease development^63^.

Cytokinins are a group of phytohormones and are involved in the regulation of various processes in plant growth and development, including: cell division control, shoot meristem initiation, leaf and root differentiation, chloroplast biogenesis, stress tolerance, and senescence. The distribution of the various cytokinins differs significantly among plant species. Plants respond to cytokinins through a multistep phosphorelay system, consisting of sensor histidine kinase (HK) proteins, histidine phosphotransfer (HPt)/histidine-containing phosphotransfer (AHPt) proteins, and effector *Arabidopsis* response regulator (ARR) proteins. The type-B ARR transcription activators have a receiver domain (RD) followed by a Myb-like domain for DNA binding (BD domain) and the glutamine (Q)-rich domain for transcriptional activation (also known as the AD domain). The expression of some ARR genes is regulated by stress and sugars, suggesting a molecular link between cytokinins, stress, and sugar signaling. When *P*. *brassicae* infects Chinese cabbage, the root hormones may become imbalanced due to the stress. The cytokinins facilitate cell division in the shoots, resulting in hypertrophy and the formation of galls, a typical symptom of clubroot. Additionally, glucose can provide energy for *P*. *brassicae* to maintain the pathogen life cycle and initiate gall development in the roots.

With the recent advances in genome sequencing and functional genomics across many plant species, the number of defense-related genes that have been identified has significantly increased^42^. We suggest that upon *P*. *brassicae* infection, a new meristematic area is established in the roots, and this may act as a sink for host auxin, carbohydrates, nitrogen, and energy, in order to maintain the pathogen and initiate gall development^68^. In the current study, the iTRAQ-based quantitative proteomics analysis identified 5,003 proteins, and more than 487 proteins, accounted for 13.4%, were up- or downregulated, indicating that *P*. *brassicae* can strongly influence plant physiology. In addition, the interaction between the plant and *P*. *brassicae* was illustrated through differential proteome analysis, which showed that proteins may be responsible for regulating various cellular activities and processes. Proteome analysis may more directly reflect functional changes, as protein levels are not always correlated with mRNA levels measured by transcriptome analysis, due to post-translational modifications and protein turnover^69,70^.

KEGG pathway analysis has indicated that the metabolism of tryptophan and glutathione may affect the clubroot of cabbage, of which β-galactosidase (GLB1) involved in tryptophan metabolism may be an important molecule regulating clubroot disease in susceptible lines. During infection, the metabolism of cysteine and in the resistant lines was inhibited, suggesting that these biological processes are likely to be the targets associated with the infection of Chinese cabbage with *P*. *brassicae* in disease-resistant lines. S-adenosylmethionine synthase (METK1) involved in cysteine and methionine metabolism was significantly upregulated during the infection peak period in our study.

Based on the results of the differential proteomic analyses, along with relevant previously released data, it is likely that Chinese cabbage defended or resisted *P*. *brassicae*, as indicated by damage due to the induction of cysteine, methionine, and tyrosine metabolism. The changes in protein profiles in *B*. *rapa* after infection by this pathogen may improve our understanding of the molecular mechanisms involved in pathogen resistance in cruciferous crops.

The glutamine synthetase cytosolic isozymes 1–5 were upregulated in the comparisons of CM-28-ck/CM-28 and CM-28/CCR-28. Cytosolic glutamine synthetase is a key protein that enhances nitrogen use in wheat during plant development^71^. Additional studies with *Arabidopsis* have showed that dynamic regulations of high and low affinity exist, which control the GS isoform GS1 at the levels of mRNA and enzymatic activities are dependent on nitrogen availability and may contribute to the homeostatic control of glutamine synthesis in *Arabidopsis* roots^72^.

Arginine decarboxylase 2 was upregulated in nine comparisons in our study. The protein is a key catalase in the biosynthesis of polyamine (PA) to catalyze the production of putrescine (Put) from arginine (Arg)^73,74^. Caffeic acid 3-O-methyltransferase-like was downregulated in the comparison of CM-28-ck/CM-28. Lignin imparts mechanical strength to stems and trunks and hydrophobicity to water-conducting vascular elements. Dicotyledonous angiosperm lignins contain two major monomer species, termed guaiacyl (G) and syringyl (S) units. The formation of the G and S units of lignin requires the activity of *O*-methyltransferase enzymes, with caffeic acid 3-*O*-methyltransferase (COMT) being preferentially involved in the formation of S lignin in alfalfa and caffeoyl CoA 3-*O*-methyltransferase (CCOMT) involved in the formation of G lignin^75^.

## Materials and Methods

### Plants and pathogens

One resistant (“CCR”) and one susceptible (“CM”) Chinese cabbage accessions were provided by the Cruciferous Subject Group at the Institute of Horticultural Research at the Yunnan Academy of Agricultural Sciences. Both lines were highly inbred. A *P*. *brassicae* isolate was collected from a research field at Songming County, Kunming City, Yunnan Province, China.

### Plant growth conditions and inoculation with *P*. *brassicae*

CCR and CM were seeded into a 50-cellseedling tray containing a 1:1 mixture of soil and vermiculite. Plants were germinated in a phytotron, where the temperature was maintained at 25 °C. After six days, seedlings had formed two true leaves, and plants were inoculated with *P*. *brassicae* plant with 0.5 mL of a resting spore suspension of isolate E3 at a standard concentration of 107 spores mL^−1^). Water was used as a control^76^. Root samples were collected at 14, 21, 28, 35, and 42 days after inoculation (DAI) and were stored at −80 °C. Each sample included three repeats, and each repeat included 10 individuals.

### Calculating the root-shoot ratios

The vegetative tissue (above-ground) and roots (below-ground) plant parts were weighed at 14, 21, 28, 35, and 42 DAI. The root-shoot ratios were calculated by dividing the fresh weight of the roots by the fresh weight of the vegetative tissue and then multiplying by 100%. Each sample included three repeats, and each repeat included 10 individuals.

### Protein digestion

Proteins were re-dissolved in 500 mM triethylammonium bicarbonate (TEAB). The protein concentration of the supernatant was first determined using the bicinchoninic acid (BCA) protein assay, then 100 μg protein was transferred to a new tube, and the final volume was adjusted to 100 μL with a 8 M urea buffer (40 mM Tris-HCl, 5 mM ethylenediaminetetraacetic acid (EDTA), 4% 3-[(3-cholamidopropyl)dimethylammonio]-1-propanesulfonate (CHAPS), 1 mM phenylmethylsulfonyl fluoride (PMSF), and 10 mM dithiothreitol (DTT), pH 8.0). Next, 11 μL of 1 M DTT was added and samples were incubated at 37 °C for 1 h, after which they were transferred into 10 K ultrafiltration tubes (Millipore). To remove the urea, 100 mM TEAB was added to the samples, samples were centrifuged, and this procedure was performed two additional times, for a total of three times. Next, 120 μL of 55 mM iodoacetamide was added to the samples and they were incubated for 20 min in the dark at room temperature. Sequencing-grade modified trypsin (Promega, Madison, WI, USA) at a 1:50 enzyme-to-substrate ratio was used to digest proteins at 37 °C overnight.

### iTRAQ labeling

After protein digestion, the resulting peptide mixture was labeled with iTRAQ 8-Plex reagent (Sciex) following the manufacturer’s instruction. The labeled peptide samples were then pooled and lyophilized in a vacuum concentrator.

### High pH reverse phase separation

The peptide mixture was re-dissolved in buffer A (20 mM ammonium formate in water and a pH 10, adjusted with ammonium hydroxide) and then fractionated by high pH separation using the Ultimate 3000 system (Thermo Fisher Scientific, MA, USA) connected to a reverse phase column (XB Ridge C18 column, 4.6 mm × 250 mm) (Waters Corporation, MA, USA). High pH separation was performed using a linear gradient, initiated with 5% of buffer B (20 mM ammonium formate in 80% can and a pH 10, adjusted with ammonium hydroxide), which was increased 45% buffer B in 40 min. The column was re-equilibrated at the initial conditions for 15 min. The column flow rate was maintained at 1 mL/min, and the column temperature was maintained at 30 °C. Twelve fractions were collected and each fraction was dried in a vacuum concentrator for the following step.

### Low pH nano-high performance liquid chromatography-tandem mass spectrometry (HPLC-MS/MS) analysis

Fractions were re-suspended in 30 μL solvent C and 30 μL solvent D (C: water with 0.1% formic acid; D: ACN with 0.1% formic acid), separated by nano-liquid chromatography (nanoLC) and analyzed by on-line electrospray tandem mass spectrometry. The experiments were performed using an Easy-nLC 1000 system (Thermo Fisher Scientific, MA, USA) connected to a Q-Exactive mass spectrometer (Thermo Fisher Scientific, MA, USA) that was equipped with an online nano-electrospray ion source. For analysis, 10 μL peptide sample was loaded onto the trap column (Thermo Scientific Acclaim PepMap C18, 100 μm × 2 cm) with a flow of 10 μL/min for 3 min, separated on an analytical column (Acclaim PepMap C18, 75 μm × 15 cm) with a linear gradient from 3% D to 32% D in 120 min. The column was re-equilibrated at the initial conditions for 10 min. The column flow rate was maintained at 300 L/min. An electrospray voltage of 2 kV (versus the inlet) of the mass spectrometer was used.

### Bioinformatics analysis

The proteome data for six samples and nine combinations were obtained: CM-28ck/CM-28, CM-28-ck/CM-35, CM-28/CM-35, CCR-28-ck/CCR-28, CCR-28-ck/CCR-35, CCR-28/CCR-35, CM-28-ck/CCR-28-ck, CM-28/CCR-28, and CM-35/CCR-35. Tandem mass spectra were processed by PEAKS Studio version 8.5 software (Bioinfor Inc., CA, USA). PEAKS DB^53^ was set up to search the Uniprot_proteome from the *Brassica rapa* database (BRAD: http://Brassicadb.org) assuming the digestion enzyme trypsin. PEAKS DB was searched with a fragmentation mass tolerance of 0.05 Da and a parent ion tolerance of 7.0 ppm. Carbamidomethylation was specified as a fixed modification. Oxidation (M), deamidation (NQ), and acetylation (Protein N-term) were specified as variable modifications. Peptides were filtered by a 1% false discovery rate (FDR) and one unique. PEAKS Q was used for peptide and protein abundance calculations. Normalization was performed by averaging the abundance of all peptides. Median values were used for averaging. Differentially expressed proteins were filtered if their fold change was greater than 1.3 and contained two unique peptides with a statistical *P*-value (ANOVA test with Benjamini and Hochberg FDR correction) below 0.05, variance homogeneity test (*P*-value > 0.05), and normal distribution test (*P*-value > 0.05).

### Equipment and settings

Hierarchical cluster analysis was conducted using the pheatmap package (https://CRAN.R-project.org/package=pheatmap). Blast2 GO version 4 was used for functional annotation. The entire protein sequence database was analyzed by BlastP and mapped and annotated with the gene ontology database. The function of differentially expressed proteins was calculated by Fisher’s exact test in BLAST2GO. Pathway analysis was processed by KOBAS (http://kobas.cbi.pku.edu.cn/)^77^. A protein-protein interaction network and the metabolic pathways were constructed using STRING v10 (www.string-db.org)^78^.

## Supplementary information

Fig. S1.

Fig. S2.

Fig. S3.

Fig. S4.
