# Author Correction: iTRAQ-based quantitative analysis reveals proteomic changes in Chinese cabbage (*Brassica rapa* L.) in response to *Plasmodiophora brassicae* infection

**DOI:** 10.1038/s41598-020-57948-1

**Published:** 2020-01-30

**Authors:** Mei Lan, Guoliang Li, Jingfeng Hu, Hongli Yang, Liqin Zhang, Xuezhong Xu, Jiajia Liu, Jiangming He, Rifei Sun

**Affiliations:** 1 Institute of Horticultural Crops, Yunnan Academy of Agricultural Sciences, Yunnan Branch of the National Vegetable Improvement Center, Kunming, 650205 China; 2 Institute of Vegetables and Flowers, Chinese Academy of Agricultural Sciences, Zhongguancun, Nandajie No. 12, Haidian District, Beijing, 100081 China; 3 Yunnan University of Chinese Medicine, Kunming, 650500 China

Correction to: *Scientific Reports* 10.1038/s41598-019-48608-0, published online 19 August 2019

In the original version of the Article, in Figure 4, the names of the differentially expressed proteins were outdated and have now been corrected to the international gene names.

Additionally, in the Supplementary Figures S2, S3 and S4 originally published with this Article, the names of the differentially expressed proteins were incorrect.

These errors have now been corrected in the PDF and HTML versions of the article, and in the Supplementary Figure files that accompany the Article.

