# Supplementary material for: iTRAQ-based quantitative analysis reveals proteomic changes in Chinese cabbage (Brassica rapa L.) in response to Plasmodiophora brassicae infection
Source: Sci Rep. 2019 Aug 19;9:12058. doi: 10.1038/s41598-019-48608-0 (PMC6700187; doi:10.1038/s41598-019-48608-0)
Supplement: Supplementary file 1 — Fig. S1. [file 41598_2019_48608_MOESM1_ESM.pdf]

iTRAQ-based quantitative analysis reveals proteomic changes in Chinese cabbage (*Brassica rapa* L.) in response to *Plasmodiophora brassicae* infection

Mei Lan[1†], Guoliang Li[2†], Jingfeng Hu[1], Hongli Yang[1], Liqin Zhang[1], Xuezhong Xu[1], Jiajia Liu[3], Jiangming He[1*] & Rifei Sun[2*]

[1]Institute of Horticultural Crops, Yunnan Academy of Agricultural Sciences, Yunnan Branch of the National Vegetable Improvement Center, Kunming 650205, China

[2]Institute of Vegetables and Flowers, Chinese Academy of Agricultural Sciences, Zhongguancun, Nandajie No. 12, Haidian District, Beijing 100081, China

[3]Yunnan University of Chinese Medicine, Kunming 650500, China

[*]For correspondence (E-mails: hejiangming666@qq.com; sunrifei@caas.cn).

†These two authors contributed equally to this work.

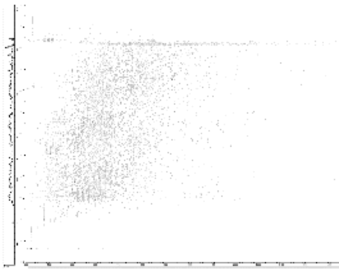
Ion Intensity Map CM-ck-1-1

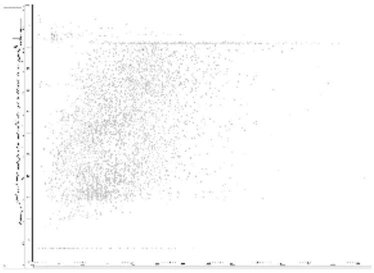
Ion Intensity Map CM-ck-1-2

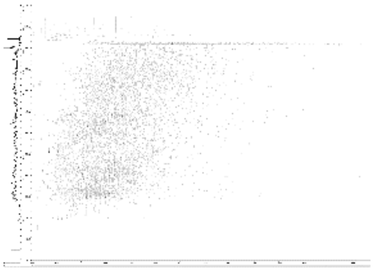
Ion Intensity Map CM-ck-1-3

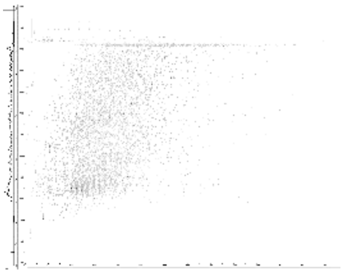
Ion Intensity Map CM-ck-2-1

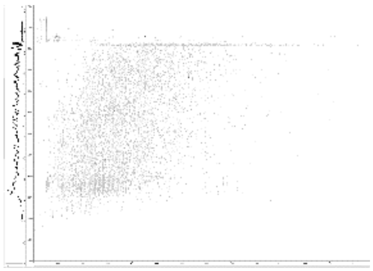
Ion Intensity Map CM-ck-2-2

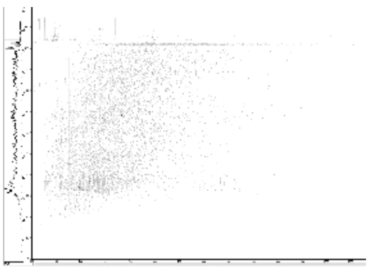
Ion Intensity Map CM-ck-2-3

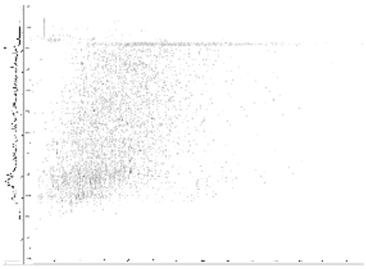
Ion Intensity Map CM-28-1-1

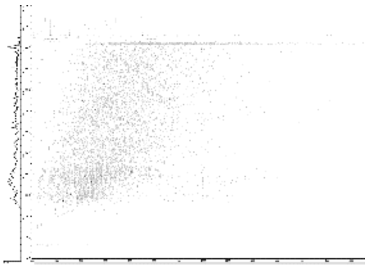
Ion Intensity Map CM-28-1-2

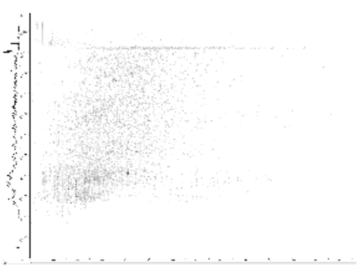
Ion Intensity Map CM-28-1-3

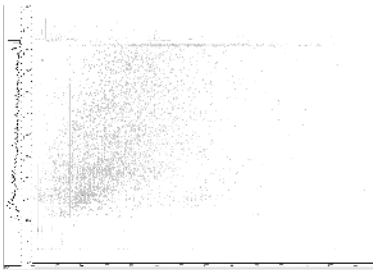
Ion Intensity Map CM-28-2-1

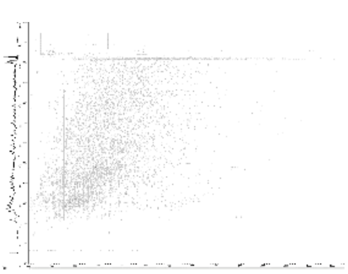
Ion Intensity Map CM-28-2-2

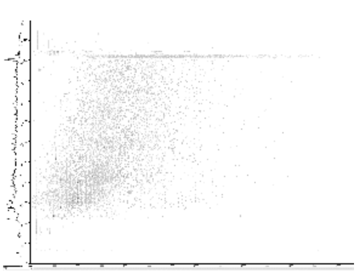
Ion Intensity Map CM-28-2-3

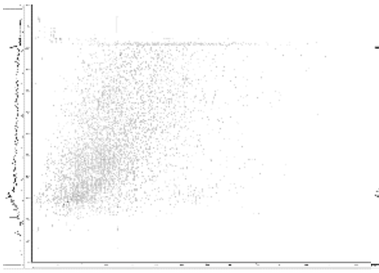
Ion Intensity Map CM-35-1-1

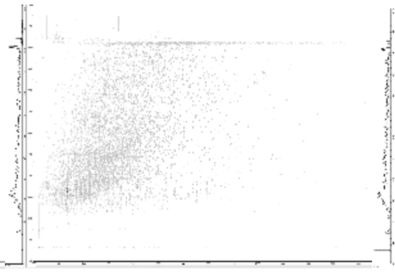
Ion Intensity Map CM-35-1-2

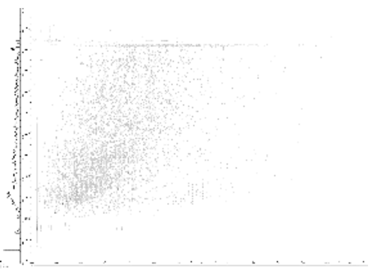
Ion Intensity Map CM-35-1-3

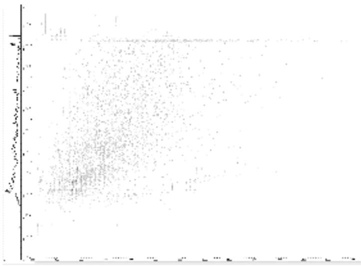

Ion Intensity Map CM-35-2-1

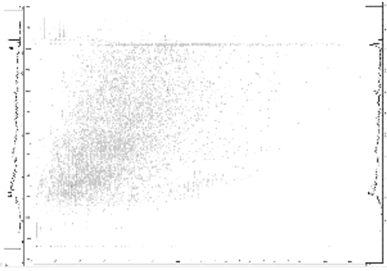

Ion Intensity Map CM-35-2-2

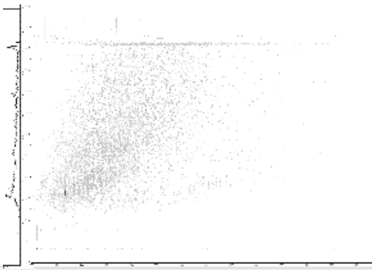

Ion Intensity Map CM-35-2-3

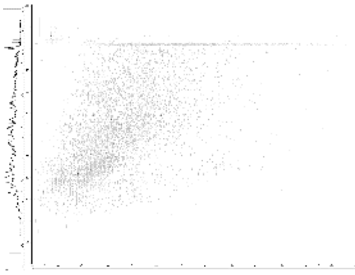

Ion Intensity Map CCR-ck-1-1

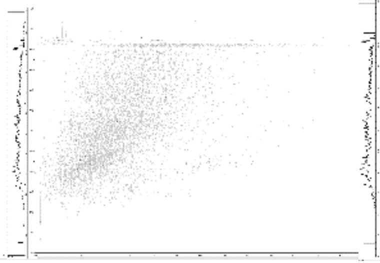

Ion Intensity Map CCR-ck-1-2

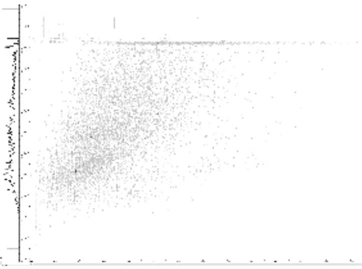

Ion Intensity Map CCR-ck-1-3

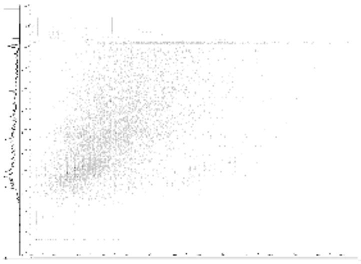

Ion Intensity Map CCR-ck-2-1

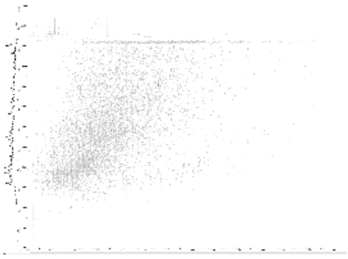

Ion Intensity Map CCR-ck-2-2

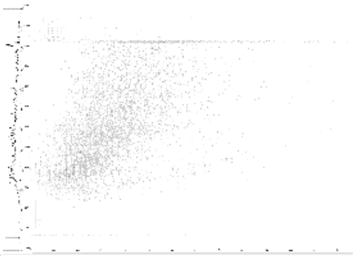

Ion Intensity Map CCR-ck-2-3

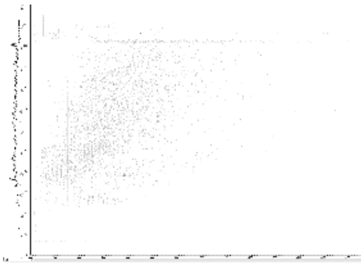

Ion Intensity Map CCR-28-1-1

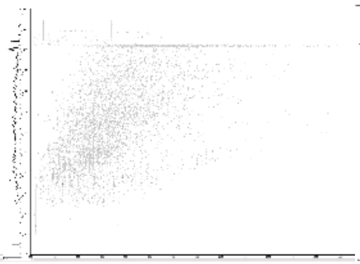

Ion Intensity Map CCR-28-1-2

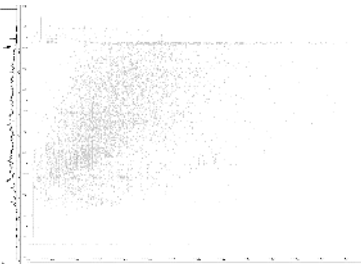

Ion Intensity Map CCR-28-1-3

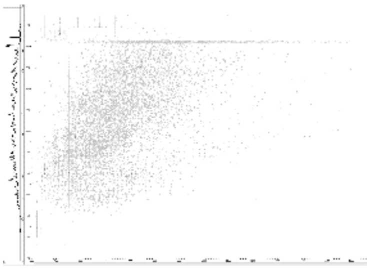

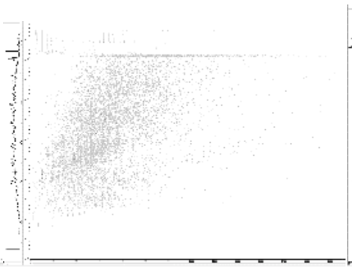

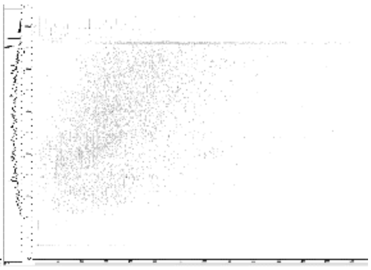

Ion Intensity Map CCR-28-2-1 Ion Intensity Map CCR-28-2-2 Ion Intensity Map CCR-28-2-3

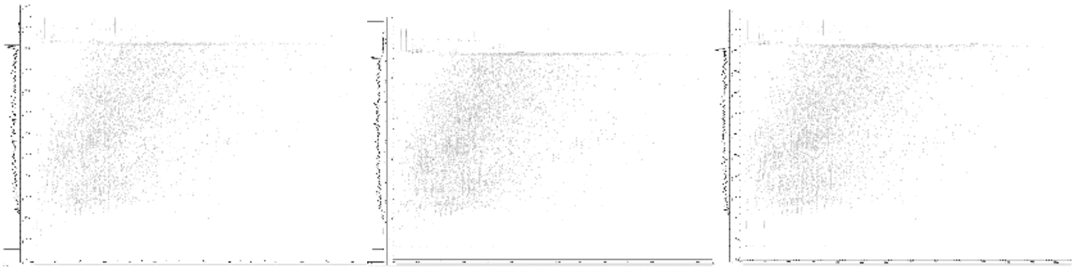

Ion Intensity Map CCR-35-1-1 Ion Intensity Map CCR-35-1-2 Ion Intensity Map CCR-35-1-3

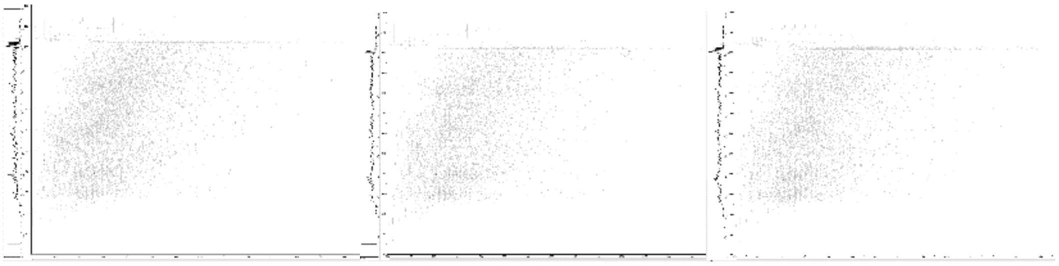

Ion Intensity Map CCR-35-2-1 Ion Intensity Map CCR-35-2-2 Ion Intensity Map CCR-35-2-3

Fig. S1. Ion Intensity Map of CM-28-ck/CM-28/CM-35/CCR-28-ck/CCR-28/CCR-35.
