# Supplementary material for: iTRAQ-based quantitative analysis reveals proteomic changes in Chinese cabbage (Brassica rapa L.) in response to Plasmodiophora brassicae infection
Source: Sci Rep. 2019 Aug 19;9:12058. doi: 10.1038/s41598-019-48608-0 (PMC6700187; doi:10.1038/s41598-019-48608-0)
Supplement: Supplementary file 2 — Fig. S2. [file 41598_2019_48608_MOESM2_ESM.pdf]

iTRAQ-based quantitative analysis reveals proteomic changes in Chinese cabbage (*Brassica rapa* L.) in response to *Plasmodiophora brassicae* infection

Mei Lan[1†], Guoliang Li[2†], Jingfeng Hu[1], Hongli Yang[1], Liqin Zhang[1], Xuezhong Xu[1], Jiajia Liu[3], Jiangming He[1*] & Rifei Sun[2*]

[1]Institute of Horticultural Crops, Yunnan Academy of Agricultural Sciences, Yunnan Branch of the National Vegetable Improvement Center, Kunming 650205, China

[2]Institute of Vegetables and Flowers, Chinese Academy of Agricultural Sciences, Zhongguancun, Nandajie No. 12, Haidian District, Beijing 100081, China

[3]Yunnan University of Chinese Medicine, Kunming 650500, China

[*]For correspondence (E-mails: hejiangming666@qq.com; sunrifei@caas.cn).

†These two authors contributed equally to this work.

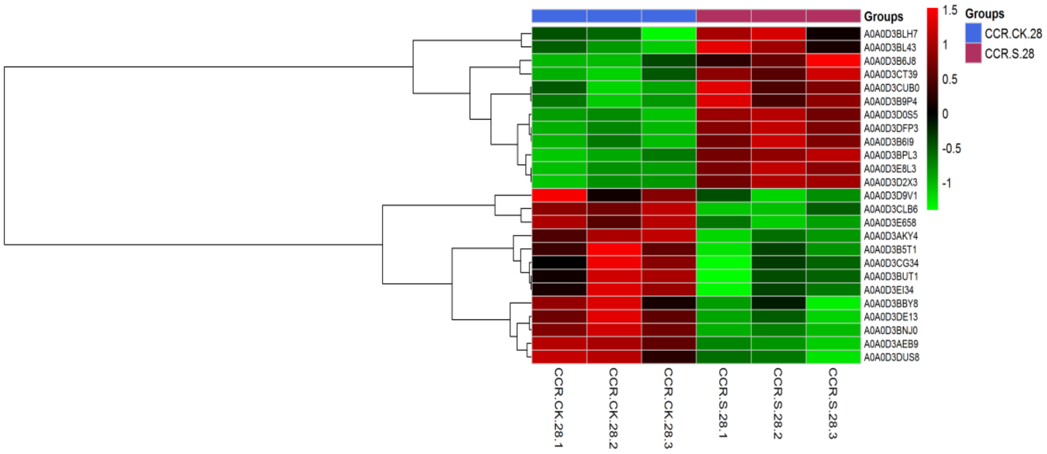

Fig. S2. Hierarchical cluster analysis between CCR-28-ck and CCR-28.
